# A CRISPR-Cas9 System for Genetic Engineering of Filamentous Fungi

**DOI:** 10.1371/journal.pone.0133085

**Published:** 2015-07-15

**Authors:** Christina S. Nødvig, Jakob B. Nielsen, Martin E. Kogle, Uffe H. Mortensen

**Affiliations:** Eukaryotic Molecular Cell Biology, Section for Eukaryotic Biotechnology, Department of Systems Biology, Technical University of Denmark, Søltofts Plads, Kongens Lyngby, Denmark; The University of Wisconsin - Madison, UNITED STATES

## Abstract

The number of fully sequenced fungal genomes is rapidly increasing. Since genetic tools are poorly developed for most filamentous fungi, it is currently difficult to employ genetic engineering for understanding the biology of these fungi and to fully exploit them industrially. For that reason there is a demand for developing versatile methods that can be used to genetically manipulate non-model filamentous fungi. To facilitate this, we have developed a CRISPR-Cas9 based system adapted for use in filamentous fungi. The system is simple and versatile, as RNA guided mutagenesis can be achieved by transforming a target fungus with a single plasmid. The system currently contains four CRISPR-Cas9 vectors, which are equipped with commonly used fungal markers allowing for selection in a broad range of fungi. Moreover, we have developed a script that allows identification of protospacers that target gene homologs in multiple species to facilitate introduction of common mutations in different filamentous fungi. With these tools we have performed RNA-guided mutagenesis in six species of which one has not previously been genetically engineered. Moreover, for a wild-type *Aspergillus aculeatus* strain, we have used our CRISPR Cas9 system to generate a strain that contains an *AACU_pyrG* marker and demonstrated that the resulting strain can be used for iterative gene targeting.

## Introduction

Filamentous fungi are ubiquitous organisms that impact human life in both positive and negative manners. For example, fungi play a major role in recirculating biomass in ecosystems, as they degrade basically all types of organic matter. For this reason they serve as a major source of industrially relevant enzymes, e.g. amylases, cellulases, lipases, pectinases, and proteases [1]. On the other hand, the same properties allow fungi to infect and deteriorate buildings, food and fodder and even living organisms, including humans, where they may cause fatal disease. Filamentous fungi also display elaborate secondary metabolisms that they use for chemical warfare, for signaling and as pigments. Many of these compounds are mycotoxins, which harm, or even kill, livestock and humans [2]. For example, several aspergilli produce carcinogenic aflatoxin and costs due to controlling mycotoxins in farm products amounts to several billion US$ per year [2,3]. However, fungal metabolites also include medically relevant compounds like antibiotic penicillins, cholesterol lowering statins and immunosuppressive mycophenolic acid [3]. Importantly, the vast majority of fungal secondary metabolites await discovery and/or medical characterization, and the pool of fungal secondary metabolites is considered a large, and still under-exploited reservoir of drug leads and potential beneficial food additives.

Despite the impact of filamentous fungi on human life, detailed knowledge into the molecular biology and biochemistry is only available for a few model fungi and there is therefore much to explore. For that reason the number of fully genome sequenced fungal species is rapidly increasing, e.g. via projects like the 1000 Fungal Genomes Project or the Aspergillus Whole-Genus Sequencing project (http://genome.jgi.doe.gov/). The fact that genetic tools are scarce or non-existent for the vast majority of fungi greatly hampers the exploitation of these genome sequences to gain experimental insights into the biology of these fungi. Firstly, lack of genetic markers makes gene engineering difficult, and secondly, if functional markers are available, low gene-targeting frequencies makes reverse genetics tedious. In model fungi, mutations disabling *pyrG*, encoding orotidine-5’-phosphate decarboxylase, offer a classic strategy to establish a robust, selectable and counter-selectable selection marker that allows for multiple rounds of gene targeting [4,5]. However, introduction of such mutations in fungi for which no genetic tools are available can be laborious and time consuming and therefore constitute a major bottleneck in the genetic characterization of fully sequenced fungi.

**Fig 1:**
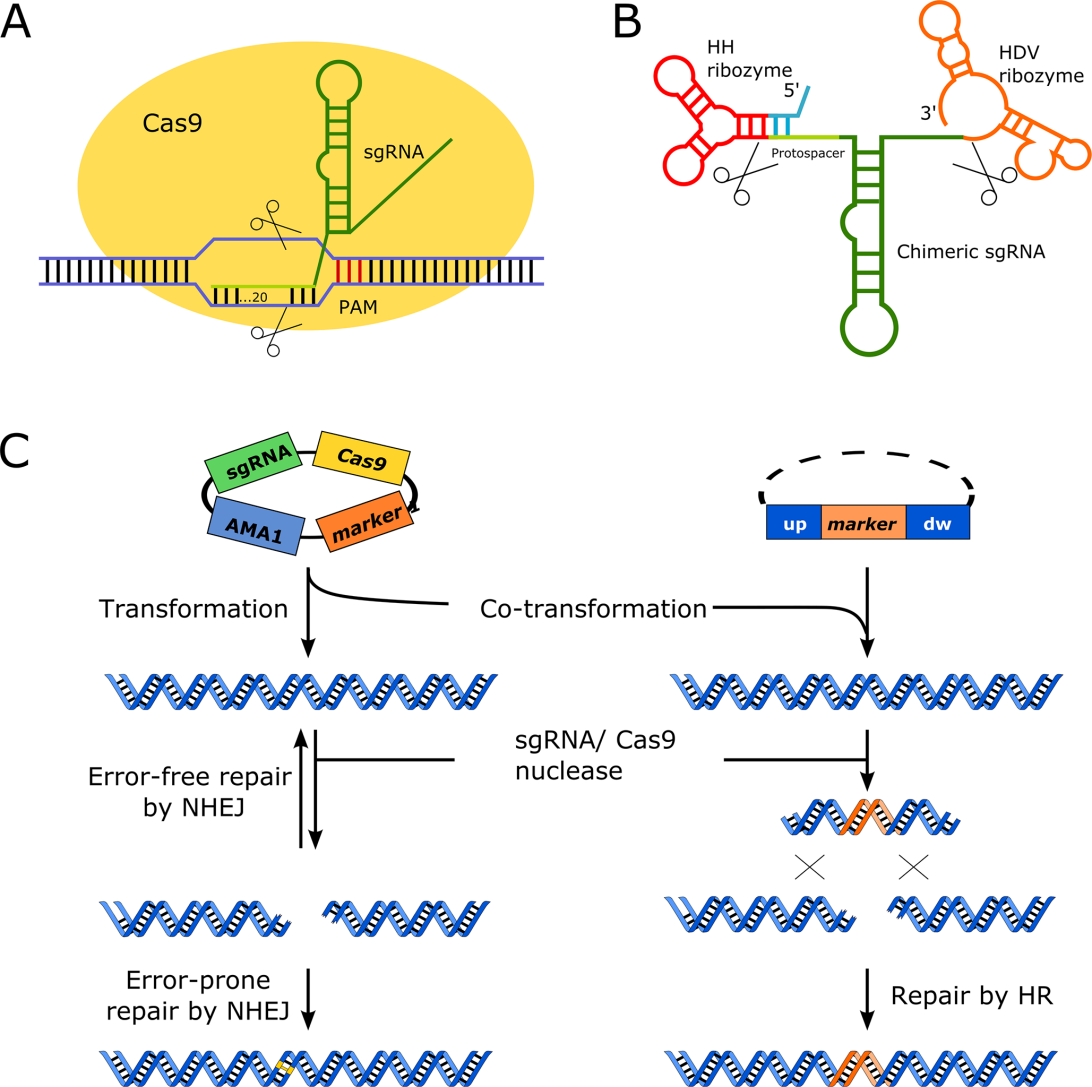
Components of the CRISPR-Cas9 system. Individual components in both panels are not drawn to scale. **A)** RNA guided nuclease Cas9 specifically cleaves a genomic target sequence. The protospacer and scaffold sections of the sgRNA are represented in light green and green, respectively. The protospacer basepairs with the complementary strand of the target sequence to form a D-loop. Note that efficient cleavage depends on the presence of the three bp PAM sequence in the target sequence, which is located directly downstream of the region invaded by the protospacer. Blunt-end cleavage occurs between bases located 3–5 bp upstream of the PAM, as indicated by scissors **B)** Liberation of the sgRNA, represented in light green/green, from a polymerase II transcript by intrinsic hammerhead (HH) ribozyme and hepatitis delta virus ribozymes (HDV) ribozyme represented in red/blue and orange respectively. Cleavage points are indicated by scissors. The blue part of HH basepairs with the protospacer for efficient cleavage. **C)** A fungal AMA1 based vector harboring Cas9 and sgRNA encoding genes are transformed into a fungus. The Cas9/sgRNA riboprotein induces a site specific DNA DSB. In the absence of a homologous template for HR repair (left lane), the DNA DSB is repaired by NHEJ; and error/prone repair by NHEJ results in mutations as indicated by yellow base pairs. In the presence of a linear or circular gene targeting substrate (right lane), specific Cas9 induced DNA DSBs may be repaired by HR resulting in a gene targeting event illustrated as insertion of an orange marker gene in this example.

The bacterial and archaeal immune mechanism CRISPR (clustered regularly interspaced short palindromic repeats)-Cas9 has recently been engineered into a powerful gene editing system [6–10]. Hence, a CRISPR-Cas9 system consisting of only two components, the Cas9 nuclease and a single chimeric guide RNA (sgRNA), allows for the introduction of specific DNA double strand breaks (DSBs), which in turn can be employed to efficiently stimulate gene targeting [11]. Accurate targeting of the RNA-guided Cas9 nuclease to a specific DNA sequence is achieved by the protospacer sequence of the sgRNA. The protospacer consists of only 20 nucleotides that recognize the target site by base pairing (Fig 1A). The fact that a synthetic oligonucleotide easily accommodates sequence corresponding to a protospacer makes it simple to construct and express genes encoding novel sgRNAs for Cas9. Programming of Cas9 to recognize new targets is therefore much easier than other systems that also function by inducing specific DSBs, such as TALENs or zinc-finger nucleases [12–14]. The only restriction of the CRISPR-Cas9 system is the requirement for a short Protospacer Adjacent Motif, PAM, adjacent to the binding site of the guiding protospacer sequence at the target site. The PAM sequence varies between Cas9 proteins [15], but for the *Streptococcus pyogenes* Cas9, which is commonly used for CRISPR-Cas9 gene editing, the very frequently occurring NGG sequences are accepted as PAMs (NAG is also accepted, but with reduced efficiency [16]. Hence, virtually all genes can be targeted by RNA-guided *Streptococcus pyogenes* Cas9. Like for TALENs and for zinc-finger nucleases, off-target effects have been reported for RNA-guided Cas9 gene editing, but a few mismatches scattered in the pairing region, especially in the 12 PAM proximal nucleotides of the protospacer, appears to eliminate mutagenesis [16,17]. Hence, off-target effects may mostly be problematic with organisms possessing large genomes. Indeed, whole genome sequencing of *Saccharomyces cerevisiae* strains that has been mutated by CRISPR-Cas9 indicated that off-target effects are insignificant in this species [18,19].

In higher eukaryotes, such as mammalian cell system or plants, where gene targeting is notoriously difficult, the CRISPR-Cas9 system has already had a huge impact and CRISPR-Cas9 has been successfully adapted to a wide range of organisms, such as yeast [20], mammalian cells [6,8], fish [7], and plants [21]. Here we present a CRISPR-Cas9 system adapted for filamentous fungi, which we have used to introduce specific mutations in six different species in two different sections of *Aspergillus*. For example, we have shown that it is possible to start with a wild-type *Aspergillus aculeatus* strain and quickly introduce an *AACU_pyrG* mutation to allow for iterative gene targeting. We envision that these tools can be used to rapidly expand the repertoire of fungi where genetic engineering is possible and therefore contribute to accelerate the exploration and industrial exploitation of fungal biology.

## Results

### A versatile CRISPR-Cas9 system for genetic engineering of filamentous fungi

**Fig 2:**
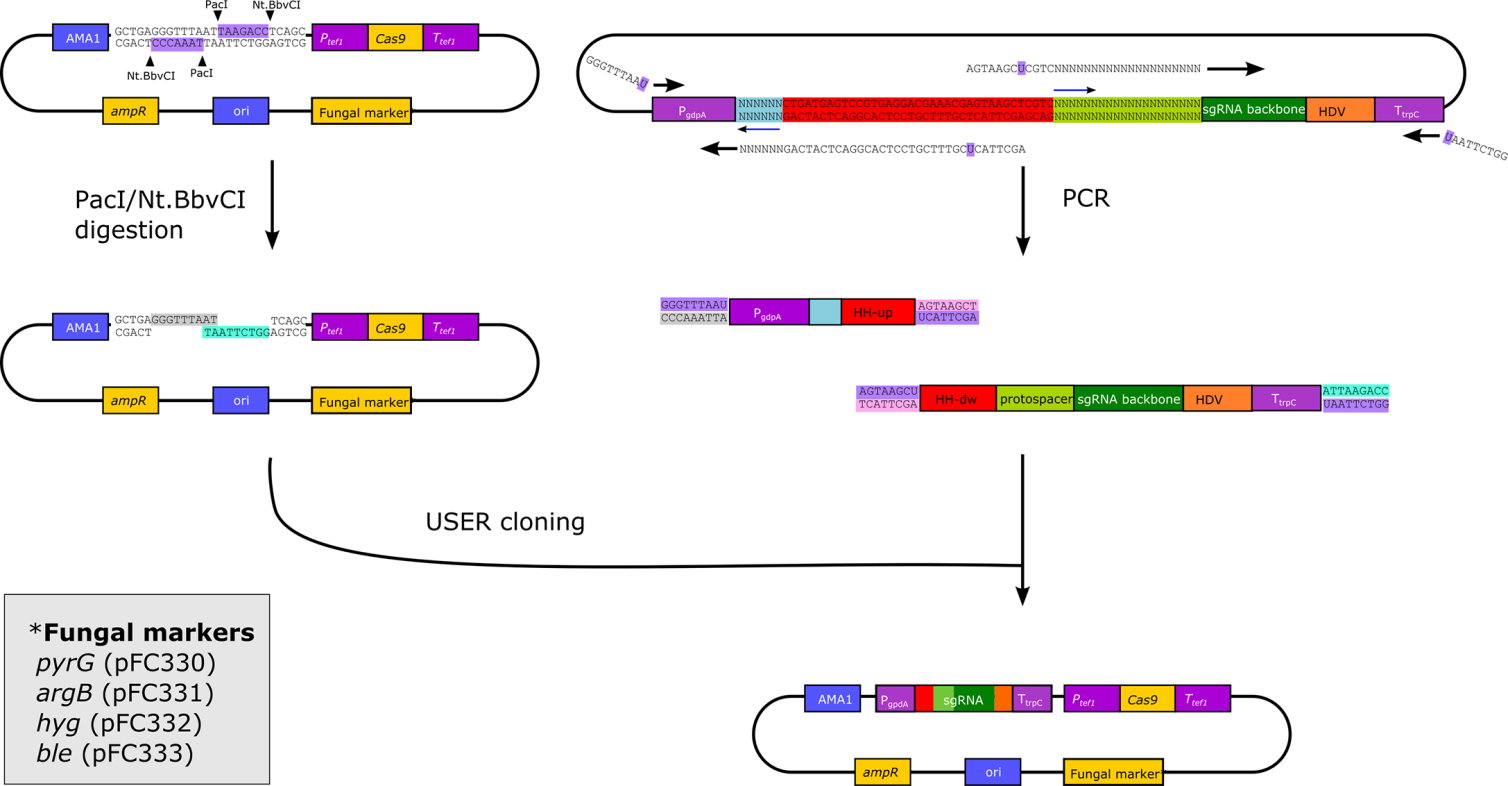
Construction of new CRISPR-Cas9 vectors for directed mutagenesis of filamentous fungi. Construction of fungal CRISPR-Cas9 vectors with variable sgRNA genes controlled by *gpdA* promoter and *trpC* terminator (no DNA elements are drawn to scale). The vector backbone for construction of new Fungal vectors for Cas9 induced genetic engineering are derived from the plasmid series pFC330-333. Sticky ends for USER cloning are achieved by opening the PacI/Nt.BbvCI USER cassette of pFC330-333 by the concerted action of restriction enzymes PacI and Nt.BbvCI (left side of panel). The two PCR fragments necessary for construction of the sgRNA gene, are both obtained by using pFC334 as template (right side of panel). This vector contains a protospacer for targeting yA (in light green), which is represented by 20 Ns indicating that it is not intended to match the primer; and in principle could be any sequence. The sections of the sgRNA gene encoding the variable parts of the transcript, the 20 bases of the protospacer (in light green) and the reverse complementing 6 bases of HH (in light blue), are introduced via tails added to the ends of the two primers that define the down- and upstream ends of the two PCR fragments, respectively. The position of the resulting inverted repeat located in the variable regions is indicated by blue arrows (top of panel). After amplification, the two PCR fragments are fused and inserted into vector pFC330-333 (Four variants exist) by USER cloning in a single step. For this purpose, each PCR fragment is generated by primers containing a tail with a uracil base (in purple). Elimination of the uracil bases in the PCR fragments by Uracil DNA glycosylase and DNA glycosylase-lyase Endonuclease VIII (USER Enzyme) results in the production of pairwise complementary overhangs at the ends of all fragments allowing selected ends to be fused in a directional manner. For simplicity, all complementary ends are visualized in the same color.

Our system for CRISPR-Cas9 mediated mutagenesis is designed for simple and versatile use in a broad spectrum of fungi. Hence, it allows directed mutagenesis by transforming a target host with a single plasmid that contains the genes encoding the two components of the CRISPR-Cas9 system, the Cas9 nuclease and a single chimeric guide RNA, see F 1. RNA guided DNA DSBs generated by Cas9 may be repaired in an error-prone manner by non-homologous end-joining (NHEJ) and directly lead to mutation; or alternatively, if a gene-targeting substrate is co-transformed into the strain together with the CRISPR-Cas9 vector, the DSBs may serve to increase the gene targeting efficiency at their site (See Fig 1C). To facilitate RNA guided mutagenesis in a broad range of filamentous fungi, four different CRISPR-Cas9 plasmids (see Fig 2) have been constructed each containing a commonly used fungal marker, *AFUM_pyrG*, *AN_argB*, *ble* ^*R*^ or *hyg* ^*R*^, as well as the AMA1 sequence, which has been shown to support replication in many different fungal species [22,23].

Both components of the CRISPR-Cas9 system were modified to enhance functionality in fungi. Firstly, the *cas9* gene from *S*. *pyogenes* was codon optimized for expression in industrially relevant *A*. *niger* and 3’-extended by a sequence encoding a SV40 nuclear localization signal (PKKKRKV). The resulting synthetic gene was fused to the strong constitutive *A*. *nidulans tef1* promoter and to the *tef1* terminator[24,25]. The resulting fusion gene includes a consensus Kozak sequence [26] in front of the start codon. Secondly, the short sgRNAs do not contain a cap structure and poly A-tail, and in CRISPR-Cas9 systems they are therefore typically transcribed from genes controlled by RNA polymerase III promoters [27]. However, as these promoters are ill defined in filamentous fungi, we employed a variant of the CRISPR-Cas9 system in which the sgRNA is embedded in the middle of a larger transcript synthesized by RNA polymerase II [27]; the sgRNA is liberated from the larger transcript in the nucleus by the action of two ribozyme sequences, 5'-end hammerhead (HH) and 3'-end hepatitis delta virus (HDV), which flank the sgRNA, (Fig 1B). Robust synthesis of the transcript containing the sgRNA sequence is ensured by employing the strong constitutive *A*. *nidulans gpdA* promoter (*PgpdA*) and the *trpC* terminator (*TtrpC*). A similar strategy has previously been employed in other organisms, e.g. yeasts [27,28] and mammalian cells [29].

### Construction of CRISPR-Cas9 vectors for directed mutagenesis of filamentous fungi

CRISPR-Cas9 vectors (see Fig 2 for details) with specific sgRNA genes are generated in a single USER fusion cloning step mediated by *E*. *coli*. The CRISPR-Cas9 vectors therefore contain an *E*. *coli* origin of replication, a B-lactamase gene for selection and a PacI/Nt.BbvCI USER cassette to facilitate insertion and construction of the sgRNA gene. Novel sgRNA genes controlled by the *PgdpA* and *TtrpC* sequences are inserted into the USER cassette by combining two PCR fragments amplified from plasmid pFC334 by USER fusion. As a consequence of the ribozyme based release strategy, there are two interdependent variable regions in the sgRNA gene. One region contains the 20 base pairs (bp) of the protospacer, the other region is a 6 bp inverted repeat of the 5’-end of the protospacer, which needs to be present in the HH sequence, where it serves to complete the HH cleavage site, (Fig 1B). USER fusion allows for incorporation of the two variable regions into the sgRNA gene in a single cloning step. Specifically, this is achieved by extending the downstream primer used to generate the fragment containing the *gpdA* promoter with a 39 nucleotide long tail, and the upstream primer used to generate the sgRNA coding PCR fragment with a 32 nucleotide long tail. These two primers and the resulting PCR fragments are the only components that need to be acquired for the construction of new target-specific CRISPR-Cas9 vectors.

### CRISPR-Cas9 efficiently introduces directed mutations into the *yA* gene of *A*. *nidulans*

**Fig 3:**
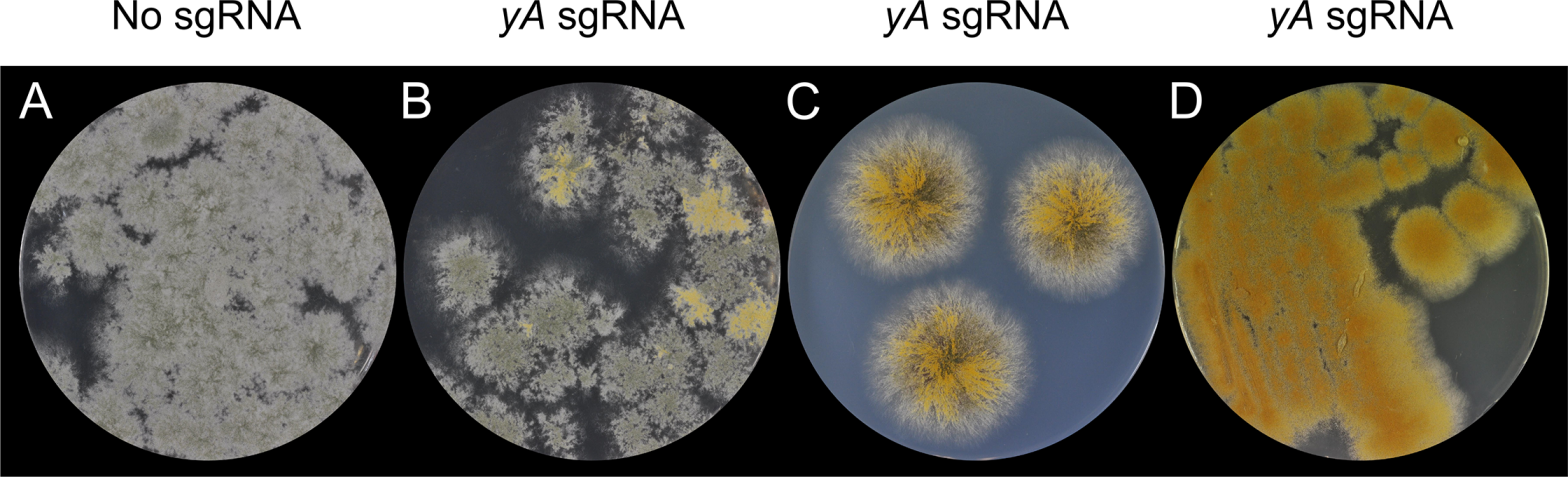
RNA guided Cas9 efficiently introduces directed mutations into the *yA* gene of *A*. *nidulans*. *A*. *nidulans* transformed with **A)** pFC331 encoding Cas9, but not a sgRNA, and with **B)** pFC334 encoding Cas9 and a *yA* specific sgRNA. **C)** Stabs of green transformants on solid selective medium from the plate shown in panel B. D) Stabs of yellow transformants on solid non-selective media. Despite loss of pFC334 they remain phenotypically stable

To test for the functionality of our system, we first attempted to mutate the *yA* gene of *A*. *nidulans*. Successful mutagenesis of this gene is easy to monitor, as inactive *yA* alleles result in a yellow color of conidia (asexual spores) rather than the green color characteristic for wild-type conidia spores [30,31]. CRISPR-Cas9 plasmids containing either no sgRNA gene, or an sgRNA gene with a protospacer targeting exon 4 of *yA* on chromosome I, were introduced into *A*. *nidulans* strain using *argB* as selection marker in three individual experiments. In the control experiments, where transformants expressed only *cas9*, the emerging colonies were all solid green (Fig 3A). In contrast, yellow colonies, or colonies with yellow sectors, were observed amongst transformants expressing both the *yA* specific sgRNA gene and *cas9* (Fig 3B) suggesting that the RNA-guided Cas9 nuclease indeed induces mutations in *yA*. As most of the colonies (approximately 70–80%) remained green on the primary transformation plates, we inoculated 12 green transformants onto solid selective medium to examine this phenotype in more detail. In all cases, each inoculation developed into yellow/green sectoring colonies with the vast majority of the colony area being yellow (Fig 3C and data not shown). This experiment shows that RNA-guided mutagenesis of *yA* is efficient in a growth dependent manner. Restreaking yellow colonies on medium without selection resulted in solid plasmid-free yellow colonies showing that the yellow phenotype is genetically stable in the absence of the *yA* specific sgRNA-Cas9 nuclease (Fig 3D). Next, to confirm that the yellow phenotype results from mutations in *yA* that are induced by the specific *yA*-sgRNA-Cas9 nuclease; we streak purified ten yellow colonies and generated PCR fragments covering the region of *yA* that was targeted by the nuclease. The ten PCR fragments were sequenced and in all ten cases mutations were identified at the site expected to be cleaved by Cas9. These results show that the mutations were likely obtained by error-prone NHEJ repair of the Cas9 induced break (Table 1). In eight out of the ten cases, small, one or two bp deletions, at the break created frameshifts in the *yA* gene explaining the lack of *yA* activity. Similarly, small indels have been reported as a frequent output of CRISPR-Cas9 mutagenesis in other species, e.g. in human HEK-293T cells [17]. The two remaining *yA* mutations contained 60 bps and 84 bps large inserts. Interestingly, these inserts are identical to two different loci on *A*. *nidulans* chromosome V. No sequences matching the protospacer-PAM sequence were identified in the regions 1 kb up- and downstream of the positions of these sequences. Moreover, the sequence with the highest identity to PAM and the adjacent 12 bps of the protospacer, which are most important for specificity, contained 7 mismatches, including one in the PAM sequence. These observations indicate that the 60 bps and 84 bps sequences were not captured due to additional RNA-guided Cas9 activity at these loci.

**Table 1:** Mutation spectrum of RNA guided Cas9 mutagenesis.

Species	Target Gene	Colony #	Mutation	Wild-Type Target Sequence^1^	Mutated Target Sequence ^2^
*A*. *nidulans*	*yA*	1, 3, 4, 8, 9, 10	1 bp deletion	GGCGGAGTATCATAACATCG	GGCGGAGTATCATAAC-TCG
	*yA*	5, 6	2 bp deletion	GGCGGAGTATCATAACATCG	GGCGGAGTATCATAA—TCG
	*yA*	7	1 bp deletion, 60 bp insertion	GGCGGAGTATCATAACATCG	GGCGGAGTATCATAAC-60 bp^4^-TCG
	*yA*	2	84bp insert	GGCGGAGTATCATAACATCG	GGCGGAGTATCATAACA-84 bp^3^-TCG
*A*. *aculeatus*	*albA*	1	1 bp deletion	CGGTTCTTCAACATGTCGCC	CG-TTCTTCAACATGTCGCC
	*albA*	2	10 bp deletion	CGGTTCTTCAACATGTCGCC	—-TTCTTCAACATGTCGCC
	*pyrG*	1	1 bp deletion	CCCACATCATCAACTGCAGCATC	ACA-CATCAACTGCAGCATC
	*pyrG*	2	2 bp deletion	CCCACATCATCAACTGCAGCATC	ACA—ATCAACTGCAGCATC
*A*. *niger*	*albA*	1	83 bp deletion	AGTGGGATCTCAAGAACTAC	50—Protospacer—13
	*albA*	2	83 bp deletion	AGTGGGATCTCAAGAACTAC	50—Protospacer—13
*A*..*carbonarius*	*albA*	1	7 bp deletion	AGTGGGATCTCAAGAACTACTGG	AGTGGGATCT———-TACTGG
	*albA*	2	24 bp deletion	AGTGGGATCTCAAGAACTACTGG	AGTGGGATCT-24bp
*A*. *luchuensis*	*albA*	1	70 bp deletion	AGTGGGATCTCAAGAACTACTGG	AGTGGGATCTCAAGAAC—70-
*A*. *brasiliensis*	*albA*	1	11 bp deletion	AGTGGGATCTCAAGAACTACTGG	AGTGGGATCT—————-GG
	*albA*	2	25 bp deletion, 1 bp insertion	AGTGGGATCTCAAGAACTACTGGATCCCCTAT	AGTG——C——————————-AT

^1^Underlined bp shows expected location for Cas9 induced DSBs.

^2^Hyphens indicate deleted bp

^3^GCCATTGTTGGCTCGTGAAGTGTACGGATTGATGTATCGTCGTATCTGCATATTGCCCCTGAGACTGATGATCATGTCTGTCGGA; match AN11611

^4^TTCAAAATCTCGGAGGCTGATTGTTCCACGATGCGGGTGACGGCTCCTCGGGGCGTTTCT; match AN10634

### *albA* and *pyrG* in *A*. *aculeatus* can be efficiently mutated by CRISPR-Cas9

**Fig 4:**
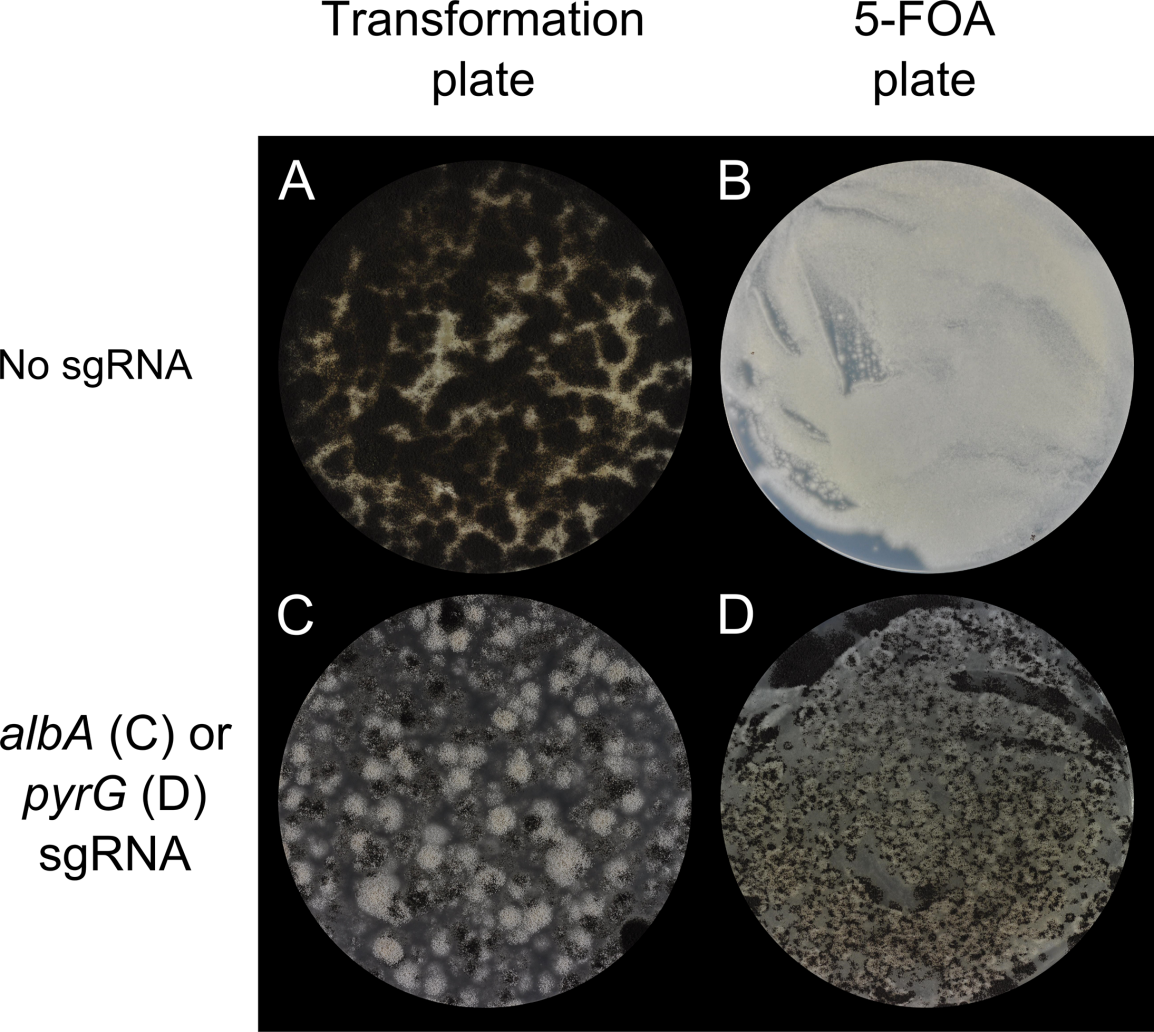
RNA guided Cas9 efficiently introduces directed mutations into *albA* and *pyrG* of *A*. *aculeatus*. *A*. *aculeatus* transformed with **A)** pFC332 encoding Cas9, but not a sgRNA plated on selective media. **B)** spores harvested from colonies shown in panel A, plated on medium containing 5-FOA, which impairs growth of *pyrG* wild-type strains preventing conidiation. **C)** pFC336 encoding Cas9 and an *albA* specific sgRNA. **D)** Spores harvested from transformants expressing Cas9 and pyrG specific sgRNA, pFC337, plated on medium containing 5-FOA. Note that colonies formed by *pyrG* mutant strains propagate normally and produce conidia.

Next, as a first test of the versatility of our fungal CRISPR-Cas9 system, we tested whether it could be used to mutagenize two different genes in *A*. *aculeatus*, 5-FOA counter-selectable *AACU_pyrG* and transcript ID 126899, which is homologous to *wA* in *A*. *nidulans* and to *albA* in *A*. *niger*. We therefore refer to 126899 as *AACU_albA* as mutation of this gene is expected to produce pale, rather than the wild-type black conidia. Accordingly, an *A*. *aculeatus* strain was transformed with CRISPR-Cas9 vectors encoding either no sgRNA, an sgRNA targeting Cas9 to the third exon of *AACU_albA* or an sgRNA targeting Cas9 to the second exon of *AACU_pyrG*. In all cases hygromycin was used for selection. As expected, transformants expressing only *cas9* formed black conidia (Fig 4A), which were unable to form colonies when plated on medium containing 5-FOA (Fig 4B). These results indicate that *AACU_albA* and *AACU_pyrG* were functional. In contrast, transformants expressing *cas9* and the specific *AACU_albA*-sgRNA gene readily formed white colonies, or colonies with white sectors (Fig 4C). Similarly, 5-FOA resistant colonies could easily be selected from a population of spores obtained from transformants expressing *cas9* and the specific *AACU_pyrG*-sgRNA gene (Fig 4D). We note that unlike mutagenesis of *yA* in *A*. *nidulans*, most colonies were solid white on the primary transformation plate suggesting that mutations occurred early after transformation. Like the *yA* mutations in *A*. *nidulans*, the *AACU_albA* mutations were genetically stable in the absence of the *AACU_albA*-CRISPR-Cas9 plasmid, as streak purified hygromycin sensitive strains produced only white conidia, see S1 Fig. Two white colonies and two 5-FOA resistant colonies were streak purified and the mutations were determined by sequencing PCR fragments covering the regions expected to be targeted by the *AACU_albA* and *AACU_pyrG* specific sgRNA. In all four cases small 1–10 bps deletions were identified at the sites expected to be cleaved by the Cas9 nuclease (Table 1). Hence, the typical RNA-guided Cas9 induced mutations in both *A*. *aculeatus* and *A*. *nidulans* appear to be small deletions. Importantly, the fact that it is possible to specifically mutate genes in *A*. *nidulans* and *A*. *aculeatus*, despite that they belong to two different sections of *Aspergillus*, show that the basic components of our CRISPR-Cas9 system may work in quite distantly related filamentous fungi.

### OPTIMuS, a script that facilitates construction of sgRNAs for targeting Cas9 to gene homologs in multiple species

The fact that the RNA guided Cas9 target site is composed of only 23 bps, i.e. the 3 bp PAM sequence and the adjacent 20 bps that match the protospacer (Fig 1A), prompted us to investigate whether it would be possible to design a protospacer, that is able to target several homologs of the same gene in different species. If so, mutation(s) could be engineered into homologs of the same gene in several species using the same vector construct. We therefore wrote a Perl script, OPTiMuS (One Protospacer for Targets in Multiple Species; see S1 OPTiMuS and S2 OPTiMUS) which facilitates identification of multi-species protospacers. Based on input FASTA files from selected species, the OPTiMuS identifies all PAM sequences in gene homologs and the accompanying protospacer sequences. Next OPTiMuS compares these protospacer-PAM sequences to identify those that are present in multiple species. Based on this analysis, the selected protospacer-PAM sequences are sorted after how many of the input sequences they may target. OPTiMuS can also be used to generate a list of protospacers, where all input sequences in a collection are matched at least once, hence, yielding a minimal set of protospacers needed to cover the collection. Additionally, as novel fully sequenced fungal genomes constantly emerge, we also included a feature in OPTiMuS that allows for a quick assessment of whether already constructed CRISPR-Cas9 vectors can be used for RNA-guided mutagenesis in the corresponding species. To test the functionality of OPTiMuS, we used AspGD [32] as a sequence resource to analyze *Aspergillus* homologs of *albA* for the presence of common protospacer-PAM sequences. The best hit was a protospacer-PAM sequence that potentially could target the gene homologs in eight species. Finally, since off-target Cas9 mutagenesis has been reported [16,17], we manually checked whether our selected protospacer-PAM sequence was prone for off-target effects. Specifically, we employed a simple BLAST search of the relevant genomes to look for sequences that match PAM and the 12 PAM proximal nucleotides of the protospacer [16,17]. Our selected *albA* protospacer-PAM sequence was not compromised by this test. Hence, it appears possible to generate sgRNAs that can target gene homologs in several different species without obvious off-target problems.

### Introduction of specific mutations in *albA* homologs in five Aspergilli using a single protospacer sequence

A CRISPR-Cas9 vector encoding the *albA*-sgRNA identified by the OPTiMuS analysis was constructed. Since Cas9 was codon optimized for *A*. *niger*, and since we have in-house experience in transforming this species, we next decided to test the potential of the *albA*-sgRNA-Cas9 complex for its ability to induce mutagenesis in the five species, *A*. *brasiliensis* (ABRA), *A*. *carbonarius* (ACAR), *A*. *luchuensis* (ALUC), *A*. *niger* (ANIG) *and A*. *tubingensis* (ATUB), which all belong to section *Nigri*. Importantly, amongst these fungi, *A*. *brasiliensis* has to our knowledge not previously been genetically engineered. The strains were therefore transformed with the *albA* specific CRISPR-Cas9 vector, and its ability to stimulate specific RNA-guided Cas9 mutations was examined. Transformants were obtained for all species except *A*. *tubingensis*. With *A*. *brasiliensis* and *A*. *niger*, white transformants, or transformants containing white sectors, readily developed on the primary transformation plate (data not shown). For *A*. *carbonarius*, a few small white sectors developed amongst approximately 50 primary transformants and for *A*. *luchuensis* a single white colony was obtained after restreaking four of the primary transformants. The single white *A*. *luchensis* transformant, two white colonies/sectors from *A*. *brasiliensis*, *A*. *carbonarius*, and *A*. *niger* were streak purified for further analysis. Like above, the region targeted by the *albA* specific Cas9 was PCR amplified and sequenced. In all five cases, indels, ranging from 7 bp to 83 bp were identified at the site expected to be cleaved by the Cas9 nuclease demonstrating that it is possible to generate CRISPR-Cas9 vectors with the ability to mutate homologs in several species. We note that despite that the two *A*. *niger albA* mutations were independently generated, our analysis showed that they were both identical 83 bp deletions. Perhaps a small three nucleotide ATA repeat present at the borders of the deletion promotes this type of event.

### CRISPR-Cas9 induced DNA DSBs efficiently stimulate gene targeting in *A*. *nidulans* and in *A*. *aculeatu*

Next, we investigated whether DNA DSBs induced by the *yA* or *albA* specific sgRNA-Cas9 could stimulate gene targeting at these loci in *A*. *nidulans* and in *A*. *aculeatus*, respectively. We therefore co-transformed NHEJ proficient strains with linear (for *A*. *nidulans* and *A*. *aculeatus*) and circular gene-targeting substrates (for *A*. *aculeatus*) designed for deleting *yA* or *albA* in combination with either the *yA*-sgRNA- or *albA*-sgRNA-CRISPR-Cas9 vector (see Fig 1C and M&M). In these experiments, the gene-targeting substrates were composed by up- and downstream targeting sequences (approximately 2000 bp each) and by a selectable *Afl_pyrG* marker flanked by a direct repeat. Use of the *Afl_pyrG* for selection in *A*. *aculeatus* was possible due to the mutant *AACU-pyrG-1* strain created by RNA-guided mutagenesis above. The CRISPR-Cas9 plasmids were selected by an *AN_argB* marker in *A*. *nidulans* and by a *hyg* ^*R*^ marker in *A*. *aculeatus*. Finally, linear and circular gene-targeting substrates were also co-transformed with a CRISPR-Cas9 negative control vector that does not encode the *yA* or *albA* specific sgRNAs.

**Fig 5:**
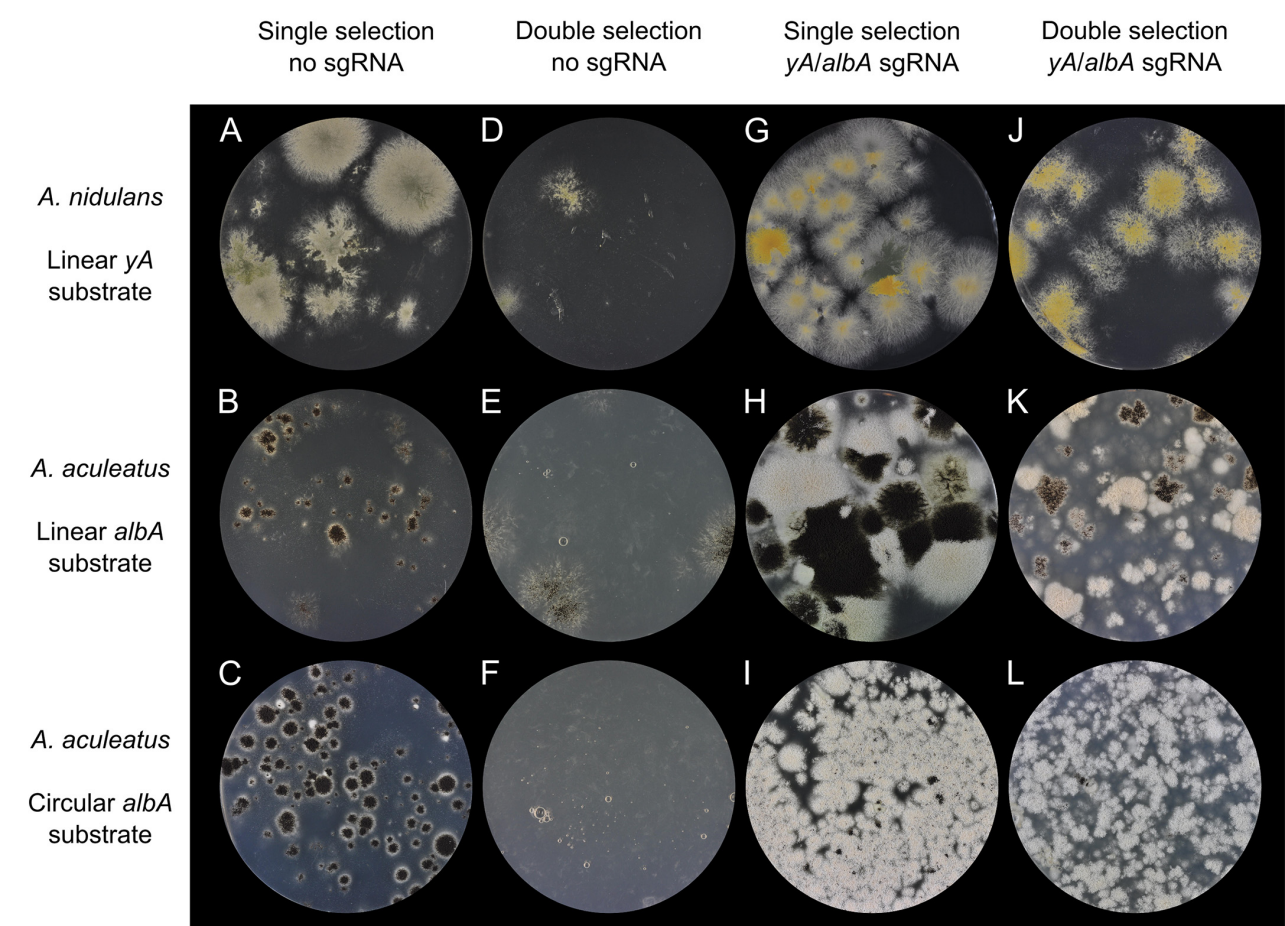
RNA guided induced DNA DSBs efficiently stimulate gene targeting in *A*. *nidulans* and *A*. *aculeatus*. *A*. *nidulans* and *A*. *aculeatus* were co-transformed with a CRISPR-Cas9 plasmid in combination with a gene targeting substrate. *yA* of *A*. *nidulans* was targeted by a linear gene targeting substrate; and *albA* of *A*. *aculeatus* was targeted by a linear as well as by a circular gene targeting substrate as indicated to the left of panels. The presence of an sgRNA gene (*yA* specific for *A*. *nidulans* and *albA* for *A*. *aculeatus*) gene in the CRISPR-Cas9-vector is indicated above panels. Selection conditions for each experiment are indicated above panels. Single selection refers to selection for the gene substrate alone; and double selection refers to selection for both the gene targeting substrate and the CRISPR-Cas9 vector.

The resulting transformants were green (*A*. *nidulans*) or black (*A*. *aculeatus*) if no RNA-guided Cas9 DNA DSBs were made at the *yA* or *albA* loci, respectively; and this was the case on plates selecting for the gene-targeting substrate (Fig 5A–5C) only, and on plates selecting for both the gene-targeting substrate and the empty CRISPR-Cas9 plasmid (Fig 5D–5F). The fact that no yellow or white mutant strains were observed likely reflects that gene targeting is inefficient in NHEJ proficient strains and that the numbers of transformants in the individual experiments are too low to produce rare transformants resulting from homologous recombination (HR).

In contrast, with the *A*. *nidulans* strain transformed with a *yA*-CRISPR-Cas9 vector and a linear *yA* gene-targeting substrate, approximately 90% of all transformants were solid yellow on both types of selection plates (Fig 5G and 5J). Pop-out recombinants, where the *AFL_pyrG* marker has been eliminated, were obtained on solid medium containing 5-FOA for eight yellow transformants from each plate. Further PCR analysis (see M&M) of these 16 strains by showed that they all contained a *yA* deletion. Four of the 16 transformants were examined in more detail to further validate that they contain a *yA* deletion. Specifically, we purified genomic DNA from two purified primary transformants and from two other transformants that were exposed to 5-FOA to eliminate the *AFL_pyrG* marker. Next, the four samples were subjected to Southern blotting using the direct repeat of the gene-targeting substrate as a probe. In agreement with the PCR analyses, the Southern blot revealed the bands that were expected if the *yA* locus has been correctly targeted in all four strains, see S2 Fig. Importantly, no other bands were observed strongly indicating that no additional copies of the gene-targeting substrate were present elsewhere in the genome.

Next, we investigated whether CRISPR-Cas9 mediated gene targeting could be performed in *A*. *aculeatus*. An *A*. *aculeatus* strain was therefore transformed with an *albA*-CRISPR-Cas9 vector and linear or circular *albA* gene targeting substrate. The fact that most of the transformants were white independently of which type of plate was used for selection (Fig 5H,5I,5K and 5L) indicated that gene targeting was efficient. To confirm that white colonies represent *albA* deletions, two white transformants from each plate were streak purified and plated on medium containing 5-FOA. Pop-out recombinants were readily obtained in all four cases and PCR analysis of the resulting strains demonstrated that they all contained the *albA* deletion. Together these results demonstrate that gene targeting is greatly stimulated by CRISPR-Cas9 induced DNA DSB breaks at the *yA* locus in *A*. *nidulans* and at the *albA* locus in *A*. *aculeatus*.

## Discussion

We have constructed a simple and versatile fungal CRISPR-Cas9 system and demonstrated its immediate potential by showing that (i) RNA-guided mutations can be introduced into specific alleles in six different species; (ii) that it is possible to use RNA-guided Cas9 induced DNA DSBs to stimulate otherwise inefficient gene targeting; and (iii) that RNA-guided mutagenesis and gene targeting can be used in succession. Importantly, the latter demonstrates that marker-free strains can quickly be engineered to allow for iterative gene targeting by e.g. mutating the endogenous *pyrG* and subsequently perform gene targeting using substrates based on *pyrG* (preferentially from a related species) as a recyclable marker. The only requirement for fungal RNA-guided Cas9 mutagenesis is the construction of target specific CRISPR-Cas9 vectors. In our version of the system this is readily achieved in a single USER-cloning based step, but other flexible cloning methods that allow for seamless joining of multiple fragments, e.g. by SLIC, Gibson DNA assembly, In-fusion or SLiCE [33–35], can easily substitute for USER-cloning if desirable. Importantly, the plasmid replicator (AMA1), the promoters, and terminators employed by the system have all been demonstrated to work in several fungal species. We therefore envision that the present version of our system, including using OPTiMuS to identify protospacers targeting the same gene, e.g. marker genes, in many different species, will speed up genetic engineering of newly sequenced fungi.

In one set of experiments, we showed that despite using an identical protospacer to stimulate RNA-guided mutagenesis, the efficiency of mutagenesis in the different species appeared to vary considerably. There may be several reasons why the mutation rate differs in individual species, but four deserve further discussion. Firstly, the *cas9* and sgRNA genes may be expressed differently in the different species due the use of fixed fungal promoters for their expression. If *cas9* and sgRNA genes are poorly expressed, RNA-guided mutagenesis may be inefficient. This problem could be alleviated by substituting the promoters in our current system by strong promoters from the host species. Similarly, the propagation stability of AMA1 based plasmids may also influence *cas9* and *sgRNA* gene expression levels, and this may also vary for the different species. In some cases, it may therefore be advantageous to integrate the *cas9*-sgRNA gene cassette into the genome of the host. Secondly, we have used a *cas9* gene that was codon optimized for efficient translation in *A*. *niger*. This may not be optimal for all species due to inefficient translation and folding of Cas9. We are currently constructing a GFP tagged version of Cas9 to quickly assess whether Cas9 levels in a new host is too low and needs to be optimized. Thirdly, since fungal protoplasts contain an unspecified number of nuclei, primary transformants are often heterokaryons. Subsequent isolation of pure homokaryotic mutant strains from the transformants is therefore frequently necessary. Pure strains are normally generated by restreaking conidia spores, but the efficiency of this process depends on the number of nuclei contained by the conidia; a number that varies from species to species. The fact that conidia from *A*. *nidulans* contain a single nucleus may explain why RNA-guided mutant strains are readily obtained in this species after one round of restreaking. In contrast, *A*. *carbonarius* and *A*. *luchuensis*, where the phenotypic development of RNA guided mutant strains required was less efficient, contain 2–5 nuclei [36]. Fourthly, the efficiency of accurate NHEJ repair may differ in individual species; and if the fidelity is high, the mutation rate will be low and depend on several cutting-sealing cycles, see Fig 1C, before the mutation is implemented. In this scenario, the mutations will develop and accumulate as a function of nuclear divisions.

We have investigated the use of RNA-guided Cas9 induced DNA DSBs to stimulate gene targeting. Traditionally gene targeting is performed using linear DNA substrates. Unlike intact circular DNA, linear DNA mimics DNA containing a DSB and will therefore recruit HR proteins to facilitate integration at a desired locus. However, linear DNA may also attract NHEJ repair proteins, which will mediate undesired random integration of the DNA fragment into the genome. With most filamentous fungi, NHEJ is dominating HR for repair and for most wild-type fungi gene-targeting efficiencies are therefore low. The fact that CRISPR-Cas9 systems introduce DNA DSB at the target locus creates a different scenario. In this case, HR proteins recruited to the target locus will use the gene-targeting substrate as a template for repair, and it can therefore be delivered either as circular or as linear DNA. We find that circular gene-targeting substrates are much more efficient for gene targeting as compared to the corresponding linear substrates. This is likely explained by the fact that intact DNA circles do not integrate into the genome by the competing NHEJ pathway, hence, reducing the number of false positives.

Although, off-target effects after CRISPR-Cas9 mutagenesis may not be a major issue for filamentous fungi, it is advisable to keep *cas9* and sgRNA expression to a minimum. Importantly, for gene targeting, we show that it is possible to achieve high efficiency in experiments where selection is applied only to the gene-targeting substrate. Since AMA1 plasmids are readily lost without selection [37], we expect that the CRISPR-Cas9 plasmid was only present during cell divisions in the early phase of mycelium formation. Another way to minimize Cas9 activity would be to equip the *cas9* gene with an inducible promoter or to increase the specificity of Cas9 induced cleavage. The latter can be achieved by employing either shorter sgRNAs [38]; or mutant Cas9 that introduces nicks rather than DNA DSBs [39]. With the latter, DNA DSB formation for mutagenesis can be achieved if two mutant Cas9 species introduce nicks in close proximity on opposite strands of the DNA molecule. Higher specificity is achieved since binding of two different sgRNAs are necessary to target two Cas9 molecules to the locus. The two approaches have been successfully used to reduce off-target effects in several organisms [38–40].

In the present paper we have introduced mutations one by one into single loci in six different fungal species. However, for mammalian cells, and for yeast, it has been shown that gene editing of several loci can be performed simultaneously [8,9,20]. Moreover, nuclease inactive Cas9 variants have been fused (or not) to transcription factor activation domains; and in combination with an sgRNA, these Cas9 species may act as synthetic transcription factor activators or repressors; and we are currently investigating the possibility of implementing similar tools for filamentous fungi. For example, we envision that Cas9 activators can be very useful for analyzing genetically silent gene clusters for secondary metabolite production. For clusters containing a transcription factor (TF) gene, the cluster may be activated by an RNA-guided Cas9 transcription factor that turns this TF gene on. For clusters without a TF gene, activation of genes in the cluster could be activated by multiplex sgRNAs that targets Cas9 transcription factors to individual genes in the cluster. If the cluster genes are successfully and specifically activated in this manner, specific combinations of multiple sgRNAs and Cas9 could be used to activate specific subsets of the genes in the cluster, which would greatly facilitate subsequent pathway elucidation. In conclusion, we show that CRISPR-Cas9 technology can be functionally adapted to work in filamentous fungi and we believe that the technology will be a major driver in forwarding the understanding of many aspects of fungal biology.

## Materials and Methods

### Strains and media

*Escherichia coli* strain DH5α was used to propagate all plasmids. The *Aspergillus* species used for implementation of CRISPR-Cas9 are listed in Table 2. Genomic DNA (gDNA) from fungal strains was isolated via FastDNA SPIN Kit for Soil DNA extraction kit (MP Biomedicals, USA). The mutant strains made in this study are listed in Table 1. All aspergilli were cultivated on standard solid glucose based minimal medium (MM) (1% glucose, 1x nitrate salt solution [41], 0.001% Thiamine, 1x trace metal solution [42], 2% agar), supplemented with 10 mM uridine (Uri), 10 mM uracil (Ura), and/or 4 mM L-arginine (Arg) when required. Solid plates containing 5-fluoroorotic acid (5-FOA) were made as MM+Arg+Uri+Ura supplemented with filter-sterilized 5-FOA (Sigma-Aldrich) to a final concentration of 1.3 mg/ml. For transformation media (TM) glucose was replaced with 1 M sucrose.

**Table 2:** Strains used in this study to implement functional CRISPR.

Species	Strain name	IBT^1^ number	genotype
*A*. *nidulans*	NID5	27263	*argB2*, *pyrG89*, *veA1*
*A*. *niger*	ATCC 1015	28639	-
*A*. *aculeatus*	ATCC 16872	3244	-
*A*. *luchuensis*	CBS 106.47	32294	-
*A*. *brasiliensis*	CBS 101740	21946	-
*A*. *carbonarius*	ITEM 5010	31236	-

^1^IBT Culture Collection, www.bio.dtu.dk; contact Ulf Thrane.

### PCR, vector construction and protospacers

All PCR products for cloning purposes were amplified in 35 cycles using proof-reading PfuX7 polymerase [43], by touch-down PCR programs with maximum annealing temperature interval ranging from 68–59°C or 64–57°C. Standard reaction volumes were 50 μl including 1x Phusion HF Buffer (New England Biolabs, USA), 0.2 mm dNTPs, 0.4 μM primers (Integrated DNA Technologies (IDT), Belgium), 1 U PfuX7, <10 ng of gDNA, 3% DMSO. PfuX7 can be substituted by Phusion U (Life Technologies). All vectors were assembled by USER cloning or USER fusion as described previously [44,45]. Vectors were constructed by USER cloning using plasmid backbones previously presented by [45]; for details concerning vector construction, see Fig 2, S1 Protocol, and S1 Table. The *cas9* gene encoding *Streptococcus pyogenes* Cas9 was codon optimized for translation in *A*. *niger* and synthetized by GeneScript in two parts, for its sequence, see genbank accession number KT031982. Sequences for the four vectors pFC330-333, and the gBlock encoding the *yA* sgRNA expression cassette, defining our present fungal CRISPR-Cas9 platform can be found at genbank with the accession numbers KT031983, KT031984, KT031985, KT031986 and KT031987. All vectors are available on request. Protospacers used to target individual fungal genes are presented in Table 3.

**Table 3:** Protospacers used in this study.

Protospacer sequence	Gene	Species
GGCGGAGTATCATAACATCG	*yA*	*A*. *nidulans*
AGTGGGATCTCAAGAACTAC	*albA*	*A*. *niger*
AGTGGGATCTCAAGAACTAC	*albA*	*A*. *brasiliensis*
AGTGGGATCTCAAGAACTAC	*albA*	*A*. *luchuensis*
AGTGGGATCTCAAGAACTAC	*albA*	*A*. *carbonarius*
GGCGACATGTTGAAGAACCG	*albA*	*A*. *aculeatus*
GATGCTGCAGTTGATGATGT	*pyrG*	*A*. *aculeatus*

### Transformation and strain validation by Tissue-PCR

Protoplastation were performed as described by Nielsen et al 2006 [31]. For transformation using either *pyrG* or *argB* as genetic marker, 10^7^ protoplasts and ~3 μg of digested DNA and 150 μl PCT solution were incubated for 10 min at room temperature, followed by adding of 250 μl ATB plating on 1 M sucrose based TM with selection. All TM plates were incubated at 30°C, except for *A*. *nidulans* transformation at 37°C. For transformation utilizing hygromycin selection, 10^7^ protoplasts and ~3 μg of digested DNA were incubated at 15 min on ice, then 1 mL PCT was added and the mix incubated for 15 min at room temperature. 100 μg/ml hygromycin B (Invivogene, USA) was added to 15 ml molten 1 M sorbitol based TM (TMsh; ~45°C), and immediately poured into an empty 9 cm petri dish. After 24 h incubation at 30°C, an overlay of 15 ml TMsh was added. All candidate transformants were streak purified prior to verification.

All strains were verified by tissue-PCR analysis using mycelium as the source of DNA. For the specific PCR protocol, see S2 Protocol, and for primers refer to S1 Table. Primers for gene-deletion analysis were designed to bind up- and downstream outside the region eliminated by the gene-targeting substrate. This setup detects wild-type sequences present in transformants that were for false positive and heterokaryons as well as for gene-deletion strains derived after marker elimination by direct repeat recombination. Transformants where a deleted gene was replaced by a marker gene was validated in two PCR reactions using a primer pair where one primer binds outside the upstream targeting region and the other binds inside the marker gene; and a pair where one of the primes bind inside the marker gene and the other binds outside of the downstream targeting region. For the experimental design and details of protocol, see S2 Protocol. Wild-type gDNA, as well as wild-type mycelium, was always included as controls to benchmark tissue-PCR efficiency for wild-type reaction success. All primers are listed in S1 Table.

### Note added in proof

While this manuscript was in revision, a functional CRISPR-Cas9 system for gene editing of *Trichoderma reesei* was published [46] demonstrating that this method may work more widely in filamentous fungi.

## Supporting Information

S1 Fig White *A*. *aculeatus* mutant strains propagate in a phenotypically stable manner. (PPTX)

S2 Fig Southern blot analysis of the *yA* locus after CRISPR-Cas9 mediated gene targeting. (PPTX)

S1 OPTiMuS Users guide to OPTiMuS. (DOCX)

S2 OPTiMuS Perl script file for OPTiMuS. (PL)

S1 Protocol Vector construction. (DOCX)

S2 Protocol PCR analysis and protocol. (DOCX)

S1 Table Primer used in this study. (DOCX)
