# Supplementary material for: A CRISPR-Cas9 System for Genetic Engineering of Filamentous Fungi
Source: PLoS One. 2015 Jul 15;10(7):e0133085. doi: 10.1371/journal.pone.0133085 (PMC4503723; doi:10.1371/journal.pone.0133085)
Supplement: S1 Fig — (PPTX) [file pone.0133085.s001.pptx]

## Slide 1
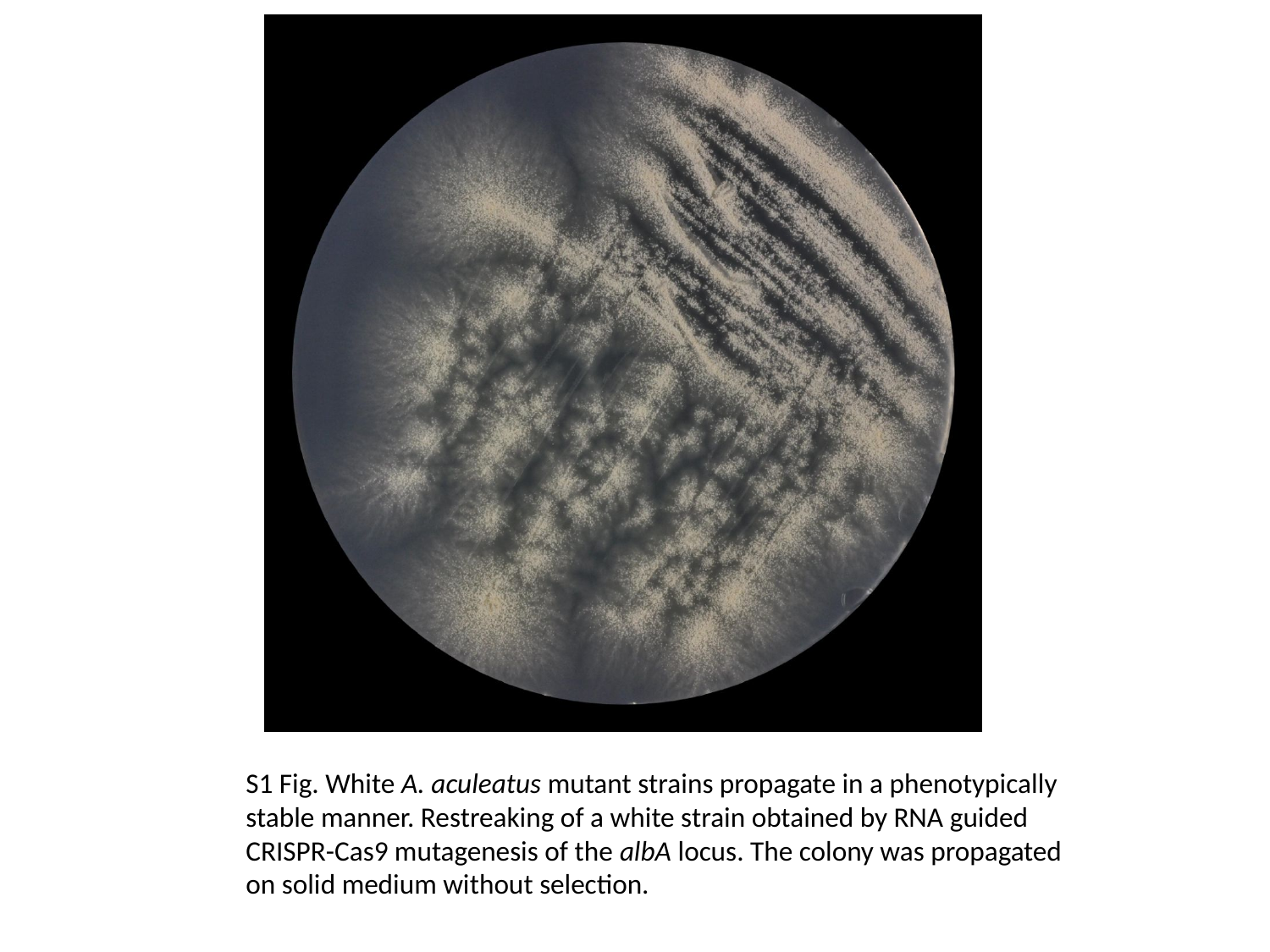

S1 Fig. White A. aculeatus mutant strains propagate in a phenotypically stable manner. Restreaking of a white strain obtained by RNA guided CRISPR-Cas9 mutagenesis of the albA locus. The colony was propagated on solid medium without selection.
