# Supplementary material for: A CRISPR-Cas9 System for Genetic Engineering of Filamentous Fungi
Source: PLoS One. 2015 Jul 15;10(7):e0133085. doi: 10.1371/journal.pone.0133085 (PMC4503723; doi:10.1371/journal.pone.0133085)
Supplement: S2 Fig — (PPTX) [file pone.0133085.s002.pptx]

## Slide 1
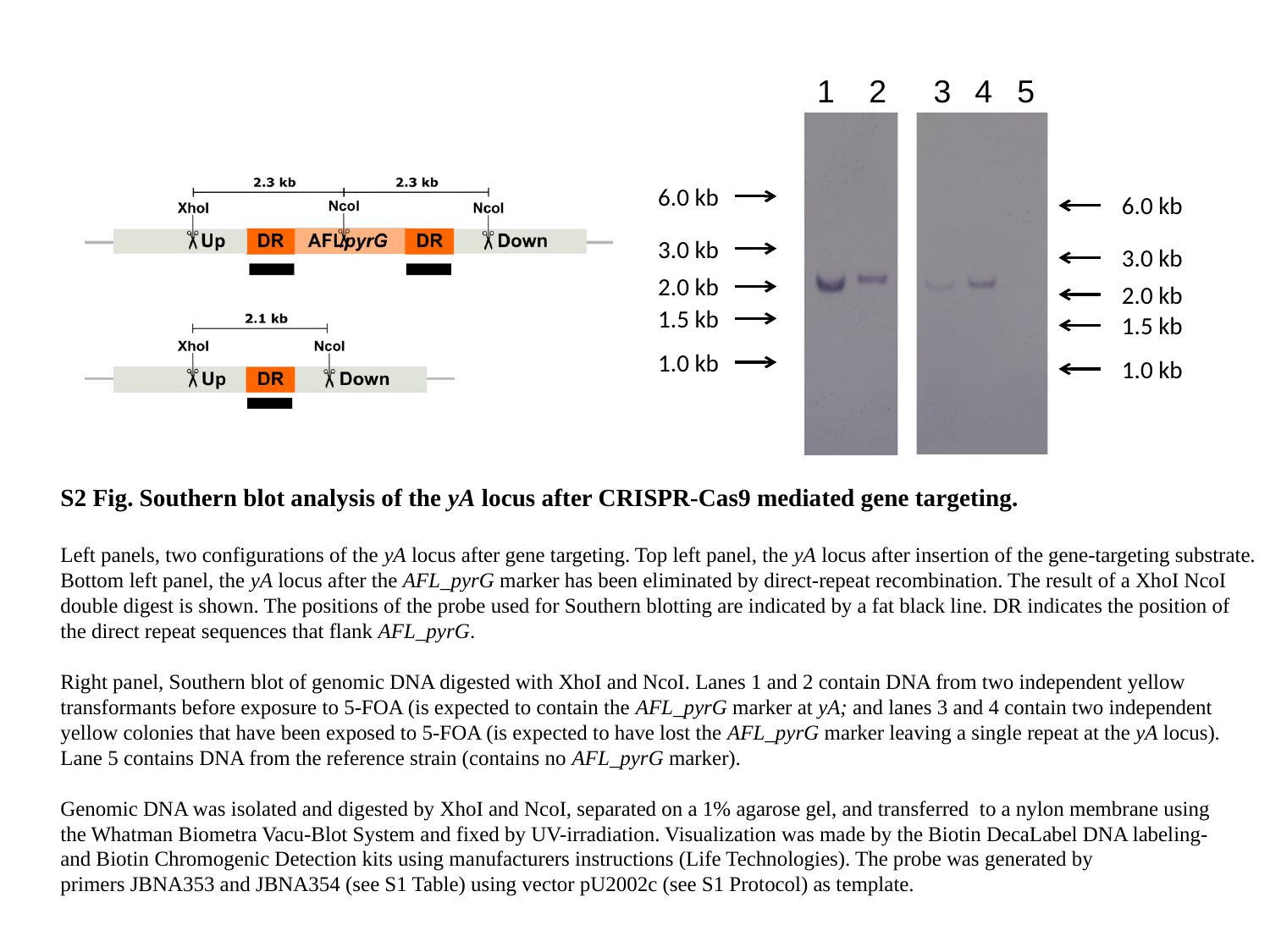

1
2
3
4
5
6.0 kb
3.0 kb
2.0 kb
1.5 kb
1.0 kb
6.0 kb
3.0 kb
2.0 kb
1.5 kb
1.0 kb
S2 Fig. Southern blot analysis of the yA locus after CRISPR-Cas9 mediated gene targeting.
Left panels, two configurations of the yA locus after gene targeting. Top left panel, the yA locus after insertion of the gene-targeting substrate.
Bottom left panel, the yA locus after the AFL_pyrG marker has been eliminated by direct-repeat recombination. The result of a XhoI NcoI
double digest is shown. The positions of the probe used for Southern blotting are indicated by a fat black line. DR indicates the position of
the direct repeat sequences that flank AFL_pyrG.
Right panel, Southern blot of genomic DNA digested with XhoI and NcoI. Lanes 1 and 2 contain DNA from two independent yellow
transformants before exposure to 5-FOA (is expected to contain the AFL_pyrG marker at yA; and lanes 3 and 4 contain two independent
yellow colonies that have been exposed to 5-FOA (is expected to have lost the AFL_pyrG marker leaving a single repeat at the yA locus).
Lane 5 contains DNA from the reference strain (contains no AFL_pyrG marker).
Genomic DNA was isolated and digested by XhoI and NcoI, separated on a 1% agarose gel, and transferred to a nylon membrane using
the Whatman Biometra Vacu-Blot System and fixed by UV-irradiation. Visualization was made by the Biotin DecaLabel DNA labeling-
and Biotin Chromogenic Detection kits using manufacturers instructions (Life Technologies). The probe was generated by
primers JBNA353 and JBNA354 (see S1 Table) using vector pU2002c (see S1 Protocol) as template.
