# Supplementary material for: A CRISPR-Cas9 System for Genetic Engineering of Filamentous Fungi
Source: PLoS One. 2015 Jul 15;10(7):e0133085. doi: 10.1371/journal.pone.0133085 (PMC4503723; doi:10.1371/journal.pone.0133085)
Supplement: S1 OPTiMuS — (DOCX) [file pone.0133085.s003.docx]

**S1 OPTiMuS. Users guide to OPTiMuS**

Optimus is designed to facilitate protospacer construction. Specifically it can perform three different tasks:

Task 1 “threshold”: Optimus can identify common protospacers in a set of selected sequences. A threshold value needs to be entered. The chosen threshold value defines the minimum number of common protospacers that exist in a set of input sequences that will be displayed. All protospacer(s) that are common in a number of sequences equal to, or above, the threshold value will be identified and sorted. The protospacers that are common in the highest number of sequences in the set will be displayed first. For example, if a set of ten input sequences is analyzed by this feature, and a threshold value of 10 is entered, OPTiMuS will show all protospacers that are common in all ten sequences if any. If a threshold value of 5 is entered, OPTiMuS will show all protospacers that are common in five, or more, of the ten input sequences (if any exist).

Task 2 “setcover”: Optimus can identify a set of protospacers that covers an entire collection of input sequences. “setcover” provides a list of protospacers covering all the input sequences, by the use of a greedy algorithm. As a result, it keeps picking the protospacer matching the most unmatched sequences until the whole set of sequences is covered. Note this does not necessarily provide the optimal global solution.

Task 3 “find”: Optimus can examine whether protospacer(s) in existing CRISPR-Cas9 vector(s) can be used to target new sequences; e.g. from newly sequenced species. “find” takes an existing protospacer and searches the input sequences for matches, taking into account the need for a PAM sequence (NGG) and all four variants of it.

**Using OPTiMuS.pl**

OPTiMuS.pl runs from a command-line interface. It uses a single FASTA file containing multiple DNA sequences and accepts files originating from Windows/UNIX/IOS.

To run the script, type the following command and follow directions:

>perl S2_OPTiMuS.pl

Alternatively, all arguments can be entered directly in the command line as:

For Task 1:
>perl S2_OPTiMuS.pl filename “threshold”/threshold value

For Task 2:
>perl S2_OPTiMuS.pl filename “set cover”

For Task3:
>perl S2_OPTiMuS.pl filename “find”/protospacer sequence
