# Supplementary material for: A CRISPR-Cas9 System for Genetic Engineering of Filamentous Fungi
Source: PLoS One. 2015 Jul 15;10(7):e0133085. doi: 10.1371/journal.pone.0133085 (PMC4503723; doi:10.1371/journal.pone.0133085)
Supplement: S1 Protocol — (DOCX) [file pone.0133085.s005.docx]

**S1 Protocol. Vector Construction.**

The *cas9* gene sequence was codon optimized for translation in *A. niger* and synthesized by GeneScript in two parts. The two *cas9* fragments were amplified with primers CSN325+CSN307 and CSN308+CSN309. The tail of primer CSN309 contains additional sequence encoding SV40-NLS in frame with the C-terminus of Cas9. The *A. nidulans* putative Tef1 (AN4218) promoter (*Ptef1*) and terminator (*Ttef1*) PCR fragments were generated by primers CSN376+CSN321 and CSN322+CSN324, respectively, using *A. nidulans* gDNA as template. All four fragments were USER cloned into the vector pU0002 [1,2], and from this vector (pU0000-Cas9) pFC330-333 was constructed. The vectors pFC330-333 were assembled by USER fusion of four individual PCR fragments of which three are common for all four plasmids. The *Ptef1-Cas9-NLS-Ttef1* was PCR amplified with the primers CSN385+CSN323, using pU0000-Cas9 as template. The AMA1 element was amplified from pAC161 in two pieces, one part was made from primers KBR088+CSN386, while the other part including a 5´ part of the *amp^R^* gene was amplified by CSN379+KBR087. The fourth variable fragment contained one of the following four fungal selection markers, *AF_pyrG* (*A. fumigatus*, pFC330), *AN_argB* (pFC331), *hph^R^* (pFC332) or *ble^R^* (pFC333), together with an *E.coli* origin and the 3’ part of the *amp^R^* marker. *AF_pyrG* was amplified with primers CSN380+CSN382, using pAC161 as template, while primers CSN380+CSN381 and template pAC76 was used for *argB.* *hph^R^* and *ble^R^* were amplified using the same primer set, CSN380+CSN378, but by using templates pAC162 or pAC245, respectively.

For expression of an *AN_yA* specific sgRNA, a gBlock (IDT, Belgium), containing a *yA* specific protospacer embedded between to ribozymes[3], flanked by the *A. nidulans gpdA* promoter and *trpC* terminator, *PgpdA* and *TtrpC*, was amplified with the primers CSN389+CSN390 and cloned into pFC331 by the PacI/Nt.BbvCI USER cassette to give pFC334. Insertion was verified by AscI/SmaI double digest (New England Biolabs, USA). To exchange the protospacer, pFC334 was used as template for two PCR fragments, with the variable regions being exchanged and assembled by the primer tails (see figure 2 for details). Vectors were prepared for cloning by PacI and Nt.BbvCI digestion [1]. For the *ACU_albA* protospacer primers CSN389+CSN414 and CSN416+CSN390 were used to generate pFC335. For multispecies *albA* primes CSN389+CSN421 and CSN422+CSN390 were used to construct pFC336. For *ACU_pyrG* sgRNA primers CSN389+CSN444 and CSN445+CSN390 were used to make pFC337. For *ACU_akuA* sgRNA CSN389+CSN425 and CSN426+CSN390 were used in the assembly of pFC338.

For construction of the gene-deletion substrates, a basic vector pU2002c was constructed based on *pyrG* including native promoter and terminator flanking *pyrG* amplified from *A. flavus* gDNA with primers CSN207+CSN208. *AFL_pyrG* was flanked by a direct repeat (DR) composed of a non-coding *S. cerevisiae* CEN.PK 520 bp stretch of gDNA, allowing for marker excision by DR recombination, amplified by primers CSN203+CSN204 and CSN205+CSN206, respectively. These three fragments where cloned into the pU0002 vector backbone, introducing 2 PacI/Nt.BbvCI USER cassettes via the primer tails, hereby creating pU2002c, which could accommodate two targeting sequences. For all three gene-deletion substrates, *AN_yA*, *ACU_albA* and *ACU_akuA*, gene-flanking sequences spanning approximately 2000 bp upstream and downstream of the target gene were amplified from the respective gDNA. *AN_yA* flanks were amplified with primers CSN142+CSN143 and CSN144+CSN145, *ACU_albA* targeting sequencing were amplified with primers CSN226+CSN227 and CSN228+CSN229, and for the *ACU_akuA* deletion vector, the primers CSN125+CSN448 and CSN449+CSN460 were used. Paired and purified targeting fragments were USER cloned into pU2002c giving pU2000c-AN*yA*, pU2000c-ACU*albA* and pU2000c-*ACU_akuA*, respectively. In the case where gene-deletion plasmids were linearized prior to transformation, SwaI digestions liberated the gene-deletion constructs from their respective vector backbone. Fragments and plasmids were purified with illustra GFX PCR DNA and Gel Band Purification Kit (GE Healthcare, USA) and GenElute Plasmid Miniprep Kit (Sigma-Aldrich, USA), respectively.

1. Hansen BG, Salomonsen B, Nielsen MT, Nielsen JB, Hansen NB, Nielsen KF, et al. Versatile enzyme expression and characterization system for Aspergillus nidulans, with the Penicillium brevicompactum polyketide synthase gene from the mycophenolic acid gene cluster as a test case. Appl Environ Microbiol. 2011;77: 3044–3051. doi:10.1128/AEM.01768-10

2. Geu-Flores F, Nour-Eldin HH, Nielsen MT, Halkier B a. USER fusion: a rapid and efficient method for simultaneous fusion and cloning of multiple PCR products. Nucleic Acids Res. 2007;35: e55. doi:10.1093/nar/gkm106

3. Gao Y, Zhao Y. Self-processing of ribozyme-flanked RNAs into guide RNAs in vitro and in vivo for CRISPR-mediated genome editing. J Integr Plant Biol. 2014;56: 343–349. doi:10.1111/jipb.12152
