# Supplementary material for: A CRISPR-Cas9 System for Genetic Engineering of Filamentous Fungi
Source: PLoS One. 2015 Jul 15;10(7):e0133085. doi: 10.1371/journal.pone.0133085 (PMC4503723; doi:10.1371/journal.pone.0133085)
Supplement: S2 Protocol — (DOCX) [file pone.0133085.s006.docx]

S2 Protocol. PCR analysis and protocol.


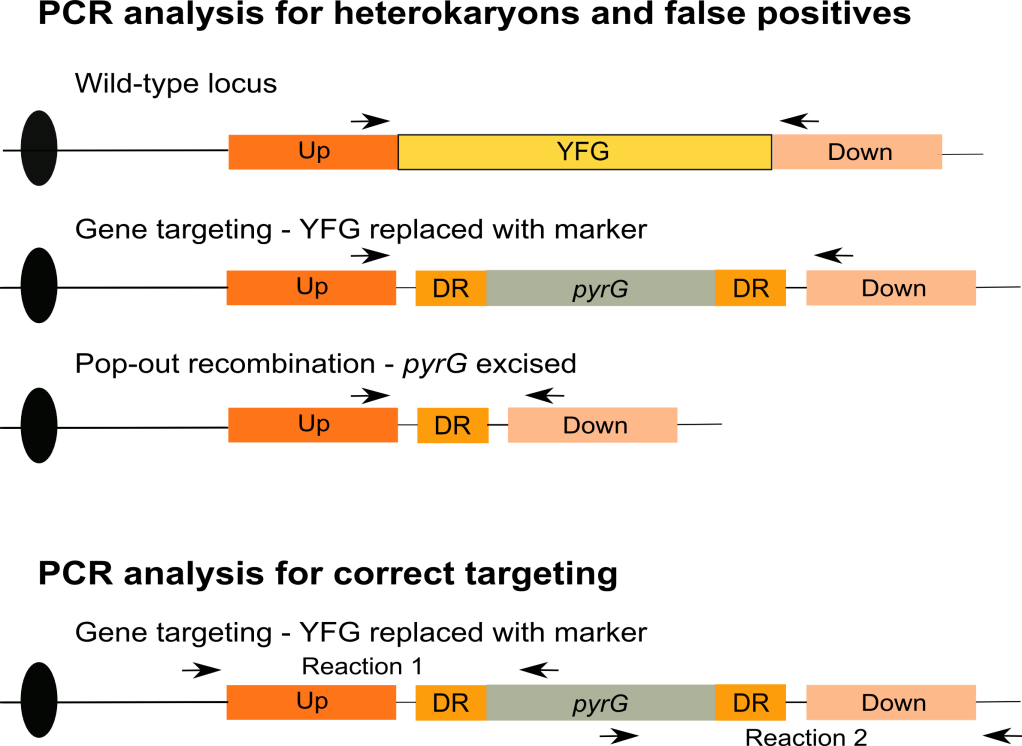


Two setups for mutant analysis were used in this study, based on PCR directly on fungal mycelium. In the upper part of the figure, our PCR analysis for heterokaryon , false positives, pop-out strains is shown. Mutant strains are always compared to wild-type strain gDNA and tissue. Depending on the locus and scenario investigated, the sizes of the three types of products vary. In the lower part, primers binding in unique locations outside the targeting sequences form primer pairs with internal primers in the marker gene. Results from both upper and lower PCR analysis were coupled for conclusion. The ease of adding mycelium directly to reaction mixture without prior purification of gDNA greatly facilitated analysis of a high number of mutants. As initial denaturation in this PCR analysis, the samples were incubated for 30 min at 98 °C to liberate genomic DNA. This treatment was followed by 35 cycles of 98 °C for 10 sec, 62 °C for 30 sec and 72 °C 3:00. Standard PCR mix in 40 µL reactions was used with following changes: template was an 1-3 mm streak of peripheral mycelium with a pipette tip, 1x Phire Reaction Buffer was added instead of HF buffer (Thermo Fisher Scientific, USA) and primer concentration was lowered to 0.25 µM. Primers that detect correct integration of the genetic marker were JBNA260+JBNA266 for *yA*, JBNA265+JBNA266 for ACU*albA*, The primers sets used for heterokaryon and pop-out check were JBNA260+JBNA261 for *yA* and JBNA263+JBNA264 for ACU*albA.* Primers for mutation analysis were binding internally in the genes to be analyzed. Thus for analyzing point mutations in *A. nidulans*, *A. brasiliensis*, *A. luchuensis*, *A. aculeatus*, *A. niger*, the following sets were used: AN*yA –* JBNM10+JBNN1, ABR*albA -* CSN465+CSN466, ALU*albA -* CSN467+CSN468, ACU*albA* - CSN474+CSN475, ASNalbA – CSN478 + CSN479. The products were purified and send for sequencing (StarSEQ GmbH, Germany).
