# Supplementary material for: A CRISPR-Cas9 System for Genetic Engineering of Filamentous Fungi
Source: PLoS One. 2015 Jul 15;10(7):e0133085. doi: 10.1371/journal.pone.0133085 (PMC4503723; doi:10.1371/journal.pone.0133085)
Supplement: S1 Table — (DOCX) [file pone.0133085.s007.docx]

**S1 Table. Primers used in this study.**

| Construction of deletion vector backbone (pFC) | | | |
| --- | --- | --- | --- |
| Backbone part 1 | CSN203 | R1-fwd | GGGTTTAAUTAAGTCCTCAGC CAAACATCTACACAATTAGCAAGGG |
| Backbone part 1 | CSN204 | R1-(L1)-rv | acgaagtcu GTTACACGGAAGGAGAGCAGTAAG |
| Backbone part 2 | CSN205 | R2-(L2)-fwd | aagtctacu CAAACATCTACACAATTAGCAAGGG |
| Backbone part 2 | CSN206 | R2-rv | GGTCTTAAUTAATGCCTCAGC GTTACACGGAAGGAGAGCAGTAAG |
| AFLpyrG marker | CSN207 | AFL-pyrG-L1-fwd | agacttcgu GTAAGTGCTTCTATATTGATCCTTAGTGC |
| AFLpyrG marker | CSN208 | AFL-pyrG-L2-rw | agtagactu GCACCTCAGAAGAAAAGGATGAT |
| Construction of specific deletion vectors | | | |
| *yA*-upstream | CSN142 | ANyA-del-up-fwd | GGGTTTAAU GTCGATCTATGGTTCCACCTCAG |
| *yA*-upstream | CSN143 | ANyA-del-up-rv | GGACTTAAU CCCGGAGGAATCAAAATGACGC |
| *yA*-downstream | CSN144 | ANyA-del-dw-fwd | GGCATTAAU GTTTGGGATTCTTAGGTGAGCTC |
| *yA*-downstream | CSN145 | ANyA-del-dw-rv | GGTCTTAAU TTATTGAGGGCCAGATTCGCGG |
| *albA*-upstream | CSN232 | AACUalbA-del-up-fwd | GGGTTTAAU CGACAAGGTACAGCGGAGATG |
| *albA*-upstream | CSN233 | AACUalbA-del--up-rv | GGACTTAAU GAGCGGCGTATTCTTCACCA |
| *albA*-downstream | CSN234 | AACUalbA-del--dw-fwd | GGCATTAAU GCGAACTAGAATGACTGGATGCT |
| *albA*-downstream | CSN235 | AACUalbA-del --dw-rv | GGTCTTAAU CCCTGAAAAGCGACGCC |
| The Cas9 basic vector | | | |
| *cas9*-5’ | CSN307 | cas9-int-rv | AGGGAAUCGTCGTGAATAAGCTG |
| *cas9*-3’ | CSN308 | cas9-int-fwd | ATTCCCU GACGTTCAAGGAGGACATCCAGA |
| *cas9*-3’-NLS | CSN309 | cas9-rv | ACCTTGCGCUTCTTCTTGGGAGG GTCGCCCCCCAGTTGACTAA |
| Anid-tef1 promoter | CSN321 | AN-Ptef1-rv | ATGGTGAAGGUTGTGTTATGTTTTGTG |
| Anid-tef1 terminator-NLS | CSN322 | AN-Ttef1-fwd | AG CGC AAG GUC TGA GCGGACATTCGATTTATGCC |
| Anid-tef1 | CSN323 | AN-Ttef1-L3-rv | ATTACACTU GTATTGGGATGAATTTTGTATGCAC |
| Anid-tef1 terminator | CSN324 | AN-Ttef1-PacI-dw-rv | GGTCTTAAU GTATTGGGATGAATTTTGTATGCAC |
| Cas9-5’ | CSN325 | Cas9-up-AnPtef-fwd | ACCTTCACCAUGGACAAGAAGTATAGCATCGGG |
| Anid-tef1-promoter | CSN376 | AN-Ptef1-PacI-up-fwd | GGGTTTAAU CGAGACAGCAGAATCACCGC |
| Hph /Ble marker +partial amp^R^ | CSN378 | PtrpC-L3-fwd | AAGTGTAAU GCTAGTGGAGGTCAACACATCAATGC |
| Fungal marker + partial amp^R^ | CSN379 | ampR-int-fwd | ACATGAUCCCCCATGTTGTGCAAAAAAG |
| Partial amp^R^ + partial AMA1 | CSN380 | ampR-int-rv | ATCATGUAACTCGCCTTGATCGTTG |
| ArgB marker+ partial amp^R^ | CSN381 | argB-L3-alt fwd | AAGTGTAAU ACTAGGTAATATCGCGTGCATTCCG |
| pyrG marker + partial amp^R^ | CSN382 | pyrG-L3-alt-fwd | AAGTGTAAU TCCTCGTGTACTGTGTAAGCGCCC |
| Anid-tef1 promoter | CSN385 | Anid-Ptef-Pac-regen-fwd | AGGGTTUAATTAAGACCTCAGC CGAGACAGCAGAATCACCGC |
| Partial AMA1 | CSN386 | Ama-alt-out-pac-regen-rv | AAACCCUCAGC GGAAACAGCTATGACCATGAGATCTTCCCG |
| Partial AMA1 + amp^R^ | KBRP087 | AMA1-int-rv | ATTGGGGUACTAACATAGCCATCAAATGCC |
| Partial AMA1 | KBRP088 | AMA1-int-fwd | ACCCCAAUGGAAACGGTGAGAGTCCAGTG |
| 5’ sgRNA expression cassette | CSN389 | PgpdA-pac-up-fwd | GGGTTTAAU GCGTAAGCTCCCTAATTGGC |
| 3’ sgRNA expression cassette | CSN390 | TtrpC-short-pac-dw-rv | GGTCTTAAU GAGCCAAGAGCGGATTCCTC |
| Aacu-albA sgRNA 5’ | CSN414 | albA-RGR-rv | AGCTTACUCGTTTCGTCCTCACGGACTCATCAGGGCGAC CGGTGATGTCTGCTCAAGCG |
| Aacu albA sgRNA 3’ | CSN416 | Acu_alb-RGR-fwd | AGTAAGCUCGTCGGCGACATGTTGAAGAACCGGTTTTAGAGCTAGAAATAGCAAGTTAAA |
| MS-albA sgRNA 5’ | CSN421 | gRNA-PS4-rv | AGCTTACUCGTTTCGTCCTCACGGACTCATCAGAGTGGG CGGTGATGTCTGCTCAAGCG |
| MS-albA sgRNA 3’ | CSN422 | gRNA-PS4-fwd | AGTAAGCUCGTC AGTGGGATCTCAAGAACTACGTTTTAGAGCTAGAAATAGCAAGTTAAA |
| Aacu-pyrG sgRNA 5’ | CSN444 | gRNA-PS8-rv | AGCTTACUCGTTTCGTCCTCACGGACTCATCAG GATGCT CGGTGATGTCTGCTCAAGCG |
| Aacu-pyrG sgRNA 3’ | CSN445 | gRNA-PS8- fwd | AGTAAGCUCGTC GATGCTGCAGTTGATGATGT GTTTTAGAGCTAGAAATAGCAAGTTAAA |
| Analysis of point mutations | | | |
| internal check of yA in *A. nidulans* | JBNM10 | ANyA-int-F | GTCCCATTTCTGCATGGACC |
| internal check of yA in *A. nidulans* | JBNN1 | ANyA-int-R | CGTAGCGCGTGCAGAGAC |
| Partial *albA* of *A. brasiliensis* | CSN465 | ABR_albA-int-fwd | TTCCTTATGCGTTCCACTCCTC |
| Partial *albA* of *A. brasiliensis* | CSN466 | ABR_albA -int-rv | ACCTCGCACACATCCAGCC |
| Partial *albA* of *A. luchuensis* | CSN467 | Aluc_albA-int-fwd | CCTTATGCCTTCCATTCCTCTCAA |
| Partial *albA* of *A. luchuensis* | CSN468 | Aluc_lbA-int-rv | TCACAGACATCCAGTCCAAGTCC |
| Partial *albA* of *A. aculeatus* | CSN474 | Aacu_albA-int-fwd | AGCCTTCACACTGCTTTCAAGAA |
| Partial *albA* of *A. aculeatus* | CSN475 | Aacu_albA-int-rv | TAGACAGGAAATGCCCACGATC |
| Partial *albA* of *A. niger* | CSN478 | Anig_albA-int-fwd | GACGGTCAACTTCCTTGGCG |
| Partial *albA* of *A. niger* | CSN479 | Anig_albA-int-rv | GTCCGTCGTACTCAGGCTTGTACT |
| Partial *pyrG* of *A. aculeatus* | CSN195 | Aacu_albA-int-fwd | AATGTGACCGTCTCCGCC |
| Partial *pyrG* of *A. aculeatus* | CSN473 | Aacu_albA-int-rv | CCCCGATTTTAGTTTCCGC |
| Check primers for PCR analysis of gene deletion events | | | |
| Check primer binds upstream and outside the targeting substrates for yA | JBNA260 | ANyA-Chk-Up-F3 | GTAGGAGCGGACAGGTGTG |
| Check primer binds downstream and outside the targeting substrates for *yA* | JBNA261 | ANyA-Chk-Dw-R3 | GGAGGGGTCGAAATGGGAGG |
| Check primer binds downstream and outside the targeting substrates for *yA* | JBNA262 | ANyA-Chk-Gap-Dw-R2 | GGCTGGGAGGGTCAGAGG |
| Check primer binds downstream and outside the targeting substrates for *albA* | JBNA263 | AACUalbA-Chk-Gap-F | GTGTGCTGGTGCAGGGAG |
| Check primer binds downstream and outside the targeting substrates for *albA* | JBNA264 | AACUalbA-Chk-Gap-R | GGAGATAGGAGGAGGAAGGAAGG |
| Check primer binds downstream and outside the targeting substrates for *albA* | JBNA265 | AACUalbA-Chk-Up-F | GGCGGATTTCTGATGCCTAGAAG |
| Internal 3’ check primer in AFL*pyrG* marker gene | CSN455 | AFLpyrG-Chk-Dw-fwd | CACCCTCGGAATAGTCCTCTCG |
| Internal 5’ check primer in AFL*pyrG* marker gene | JBNA266 | AFLpyrG-Chk-Up-R | CTAGCCACACGTTTCTCCGC |
| Internal 3’ check primer in AFLpyrG marker gene | JBNA267 | AFLpyrG-Chk-int-R | CCTCGACGATACCCTCACC |
| Probe primers for Southern blot analysis | | | |
| Primer for DR amplification for use as probe in Southern blot | JBNA353 | DR2-SB-F | CAAACATCTACACAATTAGCAAGGG |
| Primer for DR amplification for use as probe in Southern blot | JBNA354 | DR2-SB-R | GTTACACGGAAGGAGAGCAGTAAG |
